# Dietary intake in 6-year-old children from southern Poland: part 1 - energy and macronutrient intakes

**DOI:** 10.1186/1471-2431-14-197

**Published:** 2014-08-03

**Authors:** Sylwia Merkiel

**Affiliations:** 1 Food and Nutrition Department of the Eugeniusz Piasecki University School of Physical Education in Poznan, Poland, Królowej Jadwigi 27/39 Street, Poznan, 61–871, Poland

**Keywords:** Children, Dietary intake, Energy, Macronutrients, Nutrition, Diet

## Abstract

**Background:**

The studies on dietary intake in Polish children are sparse and the information about dietary intake in 6-year-olds in Europe is limited. The published studies on dietary intake in children rarely provide information on the intake of animal protein, plant protein and water. The purpose of the study was to analyse energy and macronutrient intakes in 6-year-old children from southern Poland.

**Methods:**

The studied population comprised 120 children, 64 girls and 56 boys. Energy and macronutrient intakes were estimated from a three-day food record. Weight and height were measured, and body mass index was calculated.

**Results:**

Intakes of energy (kJ, kcal), plant protein (g), total fat (g), saturated fatty acids (g, % of energy, g/1000 kcal), monounsaturated fatty acids (g) and starch (g, % of energy, g/1000 kcal) were significantly higher in boys, while intakes of sucrose (% of energy, g/1000 kcal) and total water (g/1000 kcal) were significantly higher in girls. The children’s diets were characterised by excessive intake of total fat, saturated fatty acids, sucrose, and by inadequate intake of polyunsaturated fatty acids, available carbohydrates and starch.

**Conclusions:**

The observed adverse characteristics of the children’s diets are similar to those observed in the diets of children in other European countries and show the need to work out a common educational programme to improve nutrition in young European children. It is also important to provide the lacking information about the intake of animal protein, plant protein and water in young children.

## Background

Adequate dietary intake is of vital importance to children’s growth and development, not only in physiological terms but also mental and behavioural. Both excessive and inadequate intake of energy or nutrients may have detrimental influence on children’s health and predisposes to diet-related diseases, such as hypertension, atherosclerosis, obesity, osteoporosis and type 2 diabetes later in life. This means that the prevention of these diseases should start as early as in childhood [1]. According to the Institute for Health Metrics and Evaluation [2], among the risk factors for death in both men and women all over Europe, inappropriate dietary intakes rank highest, followed by high blood pressure, while ischaemic heart disease and stroke are the two most common causes of death. Therefore, screening children for energy and nutrient inadequacies is of particular relevance to public health and to preventing diet-related diseases in population.

One of the crucial periods in a child’s life is the age of six years. In Poland and some European countries, such as Estonia, Finland and Sweden, this is the last year of preschool attendance and thus the time when the child should attain the so called ‘school readiness’ in physical, mental and emotional terms [3,4]. In other European countries, such as Belgium, France, Germany, Portugal or Spain, the age of six years is the time of attending the first class at school where the child needs to cope with the new challenges in the new environment. Both to attain the school readiness and to perform well at school, adequate dietary intakes should be provided for all children.

In the literature published after the year 2000, no publications were found on dietary intakes of 6-year-old children only. Usually, 6-year-olds are included in populations of wide age ranges and the results are reported for subgroups within these populations. In Poland, only two studies reported dietary intake of children aged 6 years or less. This is a study on 3-year-old children [5] and a national study on a representative sample which included 4-6-year-old children [6]. There is more information about dietary intake of children from other European countries. The population study of children and adolescents in Great Britain, called the National Diet and Nutrition Survey of young people aged 4–18 years, reported dietary intake for the subgroup aged 4–6 years [7]. In another British population study, the National Diet and Nutrition Survey Rolling Programme 2008/2009 – 2010/2011 [8], 6-year-old children were also included, however, in a subgroup of 4-10-year-olds. In a Belgian study on children aged 2.5-6.5 years [9,10], dietary intake was presented for a subgroup of children aged 4–6.5 years. Quite narrow age ranges were applied in a Greek study on Cretan children aged 5.7-7.6 years [11] and in a Spanish study on 6-7-year-old children [12,13]. Another Spanish study, on 2-24-year-olds [14], reported dietary intake in a subgroup of 6-9-year-old children. In a French study [15], dietary intake of 5-11-year-olds was presented. Information about energy and nutrient intakes in children outside Europe include American children from the National Health and Nutrition Examination Survey for the U.S. population [16] where the widest age ranges of subgroups were applied: less than 6years and 6–11 years. In all of these studies, except for the Spanish study on 6-7-year-olds [12,13], dietary intake was presented according to gender.

What all of the abovementioned studies have in common is analysing various sets of energy and macronutrients. Only one of these studies [9] provided information about water intake, only two [6,10] reported intake of animal and plant protein and only in one study [11] nutrient density was analysed.

In the times of globalisation it is particularly important to obtain detailed information about dietary intake of children from various countries. Therefore, the aim of this study was to analyse energy and macronutrient intakes in 6-year-old children from southern Poland, including intake of animal protein, plant protein and water, as well as nutrient density.

## Methods

### Subjects

The target population for this study were all children who attended the last grade in the preschools associated with the Nowy Sącz League of Preschools and Schools Promoting Health. The aim of the League is to popularise a healthy lifestyle, including a balanced diet, according to the programme of health promotion for preschools recommended by the Polish Ministry of Education. There were eight preschools associated with the League, all of them located in Nowy Sącz and the vicinity, a mountainous region in southern Poland. The directors of all the preschools agreed to take part in the study.

Parents of all the children who attended the last grade, a total of 253 6-year-old children, were invited to take part in the study. Parents of 149 children provided written consent. Twenty eight children who suffered from diabetes, followed special diets because of food allergies or were handicapped were excluded from the analysis of the results. Also one underreporter was excluded from further analysis. The underreporter was identified using the method described in the section *Energy and macronutrient intakes*[17]. Thus, the final population comprised 120 children, 64 girls and 56 boys. There were no siblings within the studied population.

Parents filled in questionnaires on socio-demographic characteristics of the children and their families [18,19]. The study was approved by the Bioethics Committee of the Poznan University of Medical Sciences.

### Energy and macronutrient intakes

#### Data collection

Energy and macronutrient intakes in the studied children were estimated from a three-day food record completed by parents and preschool staff. The days were determined in advance and included two preschool days and one free day (Sunday). Both parents and preschool staff were instructed how to fill in the food diaries. They provided detailed information on the time of consuming each meal, food or beverage, on the way of preparing meals (recipe, ingredients, cooking methods, etc.) and portion sizes which were measured either in grams or in typical household measures. Parents were also asked to record any supplements taken by their children.

#### Dietary assessment

Energy and macronutrient intakes were calculated using the Dieta computer programme, version 4.0, worked out by the National Food and Nutrition Institute in Warsaw, Poland. This programme is the best one in Poland so far, offering the possibility to calculate intake of energy and as many as 89 nutrients. The Dieta contains food composition database based on Polish food composition tables [20]. The database includes nutritional value not only of foodstuffs, but also of typical Polish dishes. The user may modify some ingredients of a dish (for example the kind of fat/oil used for frying) depending on the type of ingredients used by the studied person. Moreover, it is possible to calculate nutritional value of any dish using the recipe provided by the studied person. The programme estimates the changes of nutritional value by calculating the losses of nutrients resulting from food processing. The database contains nutritional value of supplements which are available in Poland.

Energy intake was expressed both in kcal and in kJ for the reason of easy comparison to the results of those studies where only joules (either kJ of MJ) or only kcal were used. Total protein intake was calculated per kg of body weight using the Microsoft Excel 2010. It is important to mention that the Dieta calculates not only total protein intake but also animal and plant protein intakes. Additional calculations were performed in the Excel to obtain animal and plant protein intakes expressed as % of total protein intake. Energy from total protein, total fat and available carbohydrates was obtained from the Dieta computer programme, while energy from fatty acids, lactose, sucrose and starch was calculated using the Excel. Total carbohydrate intake calculated by the Dieta based on Polish food composition tables was derived ‘by difference’ [20]. This method of deriving total carbohydrates is still used in many countries [21]. Additionally, available carbohydrate intake was calculated as the difference between total carbohydrates and dietary fibre using the Excel. Dietary fibre intake calculated by the Dieta means dietary fibre determined using enzymatic-gravimetric method (AOAC 1990) [20]. Total water intake calculated by the programme includes both water from beverages and water from food. Nutrient densities were estimated as amounts per 1000 kcal (4185 kJ) of energy intake.

#### Underreporting of energy intake

To identify underreporters, the ratio of energy intake to predicted basal metabolic rate (EI: BMR) was computed [17]. Basal metabolic rate (BMR) was calculated using gender- and age-dependent Oxford predictive equations from weight alone, since no significant difference was reported in predicting BMR with the inclusion of height [22]. Records with EI: BMR ratios up to 1.01 for girls and 1.04 for boys were considered as not plausible measurements of the actual three-day energy intake [17]. In the studied population, one boy with EI:BMR ratio below the abovementioned cut-off value was identified and was excluded from further analysis.

#### Comparison with nutritional guidelines

Since each individual’s energy requirement depends on numerous factors [23] and the best indicator of the adequacy or inadequacy of habitual energy intake is body weight [23,24], BMI was calculated and assessed as described in the section *Anthropometric measures* in order to conclude whether energy intake was adequate. Energy intake from macronutrients as well as cholesterol intake were compared to those recommended in the prevention of diet-related diseases [25] as in the previous article [26]. Protein intake (g/kg) was compared to the Estimated Average Requirement (EAR) and dietary fibre and total water intakes – to Adequate Intake (AI) for Polish population worked out by the National Food and Nutrition Institute in Warsaw [27].

### Anthropometric measures

Weight and height were measured, and body mass index (BMI) was calculated. BMI was classified to percentile ranges on the basis of the tables provided by Kuczmarski *et al.*[28]. The percentile ranges were called using the terminology recommended by the International Obesity Task Force [29]: below the 5^th^ percentile – underweight; from the 5^th^ to the 84^th^ percentile – healthy weight; from the 85^th^ to the 94^th^ percentile – overweight; the 95^th^ percentile or above – obesity [30].

### Statistical analysis

Statistical analysis was carried out by means of the IBM SPSS Statistics computer programme, version 19 (Chicago, IL, USA). The studied population was divided according to gender. Means and standard deviations (SD) were calculated for parents’ age. For energy and macronutrient intakes, means, standard deviations, medians and standard errors (SE) were calculated. In addition, the percentages of children with nutrient intakes below or above the recommendations were calculated to investigate the prevalence of inadequate intake.

Qualitative variables were presented in contingency tables. Statistical significance was determined using Pearson’s chi-square test. Quantitative variables were first analysed using the Shapiro-Wilk statistic for testing normality. The level of significance was set at *P* ≤ 0.05. The unpaired Student’s *t* test for normally distributed variables and the non-parametric Mann–Whitney *U* test for skewed variables were used to investigate statistically significant differences. The level of significance was set at *P* ≤ 0.05.

## Results

Table 1 shows socio-demographic characteristics of the studied 6-year-old children and their families. No statistically significant differences between girls and boys were observed.

**Table 1:** Socio-demographic characteristics of the studied 6-year-old children and their families

**Variable**	**Girls (*n*** **= 64)**	**Boys (*n*** **= 56)**	**All children (*n*** **= 120)**
Mother’s age (years)	33.0 ± 5.3^1^	33.8 ± 5.5^1^	33.4 ± 5.4^1^
Father’s age (years)	36.0 ± 6.5^1^	35.9 ± 6.4^1^	36.0 ± 6.4^1^
Mother’s education			
Vocational^2^ (%)	9.5	13.2	11.2
Secondary^3^ (%)	54.0	41.5	48.3
Higher^4^ (%)	36.5	45.3	40.5
Father’s education			
Vocational^2^ (%)	31.7	31.4	31.6
Secondary^3^ (%)	39.7	39.2	39.5
Higher^4^ (%)	28.6	29.4	28.9
Family			
Two-parent (%)	92.1	90.9	91.5
One-parent (%)	7.9	9.1	8.5
Number of children in the family			
One (%)	31.7	29.1	30.5
Two (%)	49.2	52.7	50.8
Three (%)	14.3	16.4	15.3
Four (%)	3.2	1.8	2.5
Six (%)	1.6	0.0	0.8
The sequence of the child in the family			
First (%)	64.5	63.6	64.1
Second (%)	22.6	29.1	25.6
Third (%)	9.7	7.3	8.5
Fourth (%)	1.6	0.0	0.9
Sixth (%)	1.6	0.0	0.9

^1^Mean ± standard deviation.

^2^Eight years of primary school followed by three years of vocational school.

^3^Eight years of primary school followed by four years of secondary school.

^4^Eight years of primary school, four years of secondary school and three to five years of studies ending in receiving bachelor’s or master’s degree.

Table 2 presents energy intake in the studied 6-year-old children according to the percentile categories for BMI. Although these results did not reach statistical significance, it is important to mention that energy intake increased through the percentile categories, except for the 95^th^ percentile and above.

**Table 2:** Energy intake in the studied 6-year-old children according to the percentile categories for BMI

**Percentile categories for BMI**	**Energy intake (kcal)**				**Population**	
	**Mean**	**SD**	**Median**	**SE**	**%**	**n**
Below the 5^th^ percentile (underweight)	1696	188	1726	94	3.3	4
5^th^ – 84^th^ percentile (healthy weight)	1820	314	1801	35	68.3	82
85^th^ – 94^th^ percentile (overweight)	1942	354	1954	68	22.6	27
95^th^ percentile and above (obesity)	1763	237	1650	90	5.8	7

Table 3 shows energy and macronutrient intakes in the studied 6-year-old children. Intakes of energy (kJ, kcal), plant protein (g), total fat (g), saturated fatty acids (g,% of energy, g/1000 kcal), monounsaturated fatty acids (g) and starch (g, % of energy, g/1000 kcal) were significantly higher in boys, while intakes of sucrose (% of energy, g/1000 kcal) and total water (g/1000 kcal) were significantly higher in girls. It is also important to note that total fat density was higher in boys, whereas total carbohydrates density and available carbohydrates density were higher in girls, although these findings did not reach statistical significance (*P*: 0.057, 0.073 and 0.080, respectively).

**Table 3:** Energy and macronutrient intakes in the studied 6-year-old children

**Energy/nutrient**	**Reference values**	**Girls (*n*** **= 64)**		**Boys (*n*** **= 56)**		**All children (*n*** **= 120)**		***P***	**Girls (*n*** **= 64)**		**Boys (*n*** **= 56)**		**All children (*n*** **= 120)**	
		**Mean**	**SD**	**Mean**	**SD**	**Mean**	**SD**		**Median**	**SE**	**Median**	**SE**	**Median**	**SE**
Energy														
(kcal)	body weight dependent	1781	310	1907	318	1840	319	0.035	1746	39	1863	43	1812	29
(kJ)		7462	1298	7986	1332	7707	1335	0.034	7313	162	7809	178	7589	122
Total protein														
(g)	body weight dependent	59.1	10.8	62.6	12.1	60.7	11.5	0.145	57.1	1.3	60.5	1.6	58.4	1.1
(g/kg body weight)	0.84^1^	2.6	0.6	2.7	0.5	2.7	0.6	0.758	2.5	0.8	2.7	0.7	2.6	0.5
(% of energy)	10-15%	13.4	1.8	13.3	1.7	13.4	1.7	0.935	13.0	0.2	13.7	0.2	13.2	0.2
(g/1000 kcal)	NA	33.4	4.4	32.9	4.2	33.2	4.3	0.908	32.3	0.5	33.9	0.6	32.8	0.4
Animal protein														
(g)	NA	40.1	9.4	41.7	10.5	40.8	9.9	0.386	38.9	1.2	40.9	1.4	39.3	0.9
(% of total protein)	NA	67.4	5.3	66.0	6.3	66.7	5.8	0.538	68.1	0.7	68.0	0.8	68.1	0.5
(g/1000 kcal)	NA	22.7	4.6	22.0	4.5	22.3	4.6	0.929	22.1	0.6	22.8	0.6	22.3	0.4
Plant protein														
(g)	NA	19.0	3.2	20.9	3.7	19.9	3.6	0.003	18.9	0.4	20.5	0.5	19.6	0.3
(% of total protein)	NA	32.6	5.3	34.0	6.3	33.3	5.8	0.538	31.9	0.6	32.0	0.8	31.9	0.5
(g/1000 kcal)	NA	10.7	1.0	11.0	1.2	10.8	1.1	0.183	10.7	0.1	11.0	0.2	10.8	0.1
Total fat														
(g)	NA	64.7	13.7	72.2	18.2	68.2	16.3	0.026	64.5	1.7	69.0	2.4	66.1	1.5
(% of energy)	20-30%	32.2	4.1	33.3	3.8	32.7	4.0	0.062	32.2	0.5	33.5	0.5	32.7	0.4
(g/1000 kcal)	NA	36.3	4.6	37.6	4.3	36.9	4.5	0.057	36.3	0.6	37.9	0.6	37.0	0.4
Saturated fatty acids														
(g)	NA	27.87	6.82	31.98	9.14	29.79	8.21	0.006	27.89	0.85	30.64	1.22	29.13	0.75
(% of energy)	<10%	14.1	2.3	14.9	2.3	14.5	2.3	0.038	14.0	0.3	14.6	0.3	14.4	0.2
(g/1000 kcal)	NA	15.62	2.50	16.59	2.54	16.07	2.56	0.038	15.56	0.31	16.21	0.34	16.05	0.23
Polyunsaturated fatty acids														
(g)	NA	8.15	3.11	8.40	2.58	8.26	2.87	0.474	7.71	0.39	7.91	0.34	7.87	0.26
(% of energy)	6-10%	4.2	1.7	4.0	1.1	4.1	1.4	0.846	3.8	0.2	3.7	0.1	3.8	0.1
(g/1000 kcal)	NA	4.62	1.88	4.41	1.21	4.52	1.60	0.842	4.24	0.23	4.16	0.16	4.22	0.15
Monounsaturated fatty acids														
(g)	NA	23.98	5.34	26.94	7.17	25.36	6.41	0.039	23.94	0.67	25.13	0.96	24.38	0.58
(% of energy)	>10%^2^	12.1	1.7	12.6	1.8	12.3	1.8	0.108	12.1	0.2	12.4	0.2	12.2	0.2
(g/1000 kcal)	NA	13.45	1.85	14.03	2.05	13.72	1.96	0.108	13.46	0.23	13.81	0.27	13.57	0.18
Cholesterol														
(mg)	<300	269	85	277	83	273	84	0.474	244	11	260	11	257	8
(g/1000 kcal)	NA	151	41	145	33	148	37	0.621	141	5	137	4	140	3
Total carbohydrates														
(g)	NA	254.2	49.1	266.0	41.1	259.7	45.7	0.160	249.9	6.1	259.6	5.5	255.7	4.2
(g/1000 kcal)	NA	142.5	11.8	140.1	11.0	141.4	11.5	0.073	141.6	1.5	138.5	1.5	140.1	1.0
Available carbohydrates														
(g)	130^3^	239.1	47.0	250.0	38.8	244.2	43.6	0.170	233.1	5.9	243.8	5.2	240.9	4.0
(% of energy)	55-70%^4^	53.6	4.6	52.7	4.1	53.2	4.4	0.084	53.3	0.6	52.3	0.6	52.7	0.4
(g/1000 kcal)	NA	134.0	11.5	131.6	10.3	132.9	11.0	0.080	133.3	1.4	130.8	1.4	131.8	1.0
Lactose														
(g)	NA	17.1	7.4	17.3	7.3	17.2	7.3	0.975	16.5	0.9	15.3	1.0	15.9	0.7
(% of energy)	NA	3.9	1.6	3.6	1.4	3.7	1.5	0.420	3.9	0.2	3.6	0.2	3.6	0.1
(g/1000 kcal)	NA	9.6	4.0	9.1	3.5	9.4	3.8	0.420	9.7	0.5	8.9	0.5	9.1	0.3
Sucrose														
(g)	NA	83.8	25.9	82.5	19.6	83.2	23.1	0.770	81.1	3.2	82.8	2.6	82.2	2.1
(% of energy)	NA	18.6	4.1	17.4	3.5	18.0	3.9	0.035	18.7	0.5	17.5	0.5	18.2	0.4
(g/1000 kcal)	NA	46.5	10.3	43.4	8.9	45.1	9.7	0.035	46.6	1.3	43.8	1.2	45.6	0.9
Starch														
(g)	NA	111.1	20.2	124.7	21.9	117.5	22.0	0.001	108.2	2.5	126.4	2.9	114.5	2.0
(% of energy)	NA	25.1	3.0	26.3	3.0	25.6	3.0	0.030	24.6	0.4	26.0	0.4	25.3	0.3
(g/1000 kcal)	NA	62.7	7.4	65.7	7.5	64.1	7.6	0.030	61.5	0.9	65.1	1.0	63.3	0.7
Dietary fibre														
(g)	14^5^	15.2	3.0	16.0	3.6	15.6	3.3	0.166	15.1	0.4	15.8	0.5	15.2	0.3
(g/1000 kcal)	NA	8.6	1.2	8.5	1.7	8.5	1.5	0.293	8.4	0.2	8.1	0.2	8.4	0.1
Total water														
(g)	1600^5^	1478	235	1506	231	1491	233	0.515	1463	29	1492	31	1470	21
(g/1000 kcal)	NA	838	106	799	113	819	111	0.034	831	13	774	15	815	10

*P* – significance; NA – not available; NS – not significant (*P* > 0.05).

^1^EAR.

^2^Calculated by difference as: total fat – (saturated fatty acids + polyunsaturated fatty acids).

^3^RDA.

^4^Calculated by difference: as the percentage of total energy – energy from total protein – energy from total fat.

^5^AI.

Table 4 presents the percentages of the studied 6-year-old children in the reference ranges for macronutrient intake. No statistically significant differences between girls and boys were observed. However, it is noteworthy that almost all of the studied children exceeded the recommended intake of energy from saturated fatty acids and almost all of them had intakes of energy from polyunsaturated fatty acids below the recommendations.

**Table 4:** The percentages of the studied 6-year-old children in the reference ranges for macronutrient intake

**Nutrient**	**Girls (*n*** **= 64)**	**Boys (*n*** **= 56)**	**All children (*n*** **= 120)**	***P***
	**%**	**%**	**%**	
Total protein (% of energy)				
Below the recommendations	0.0	3.6	1.7	0.309
Within the recommendations	84.4	80.4	82.5	
Above the recommendations	15.6	16.1	15.8	
Total fat (% of energy)				
Within the recommendations	26.6	23.2	25.0	0.673
Above the recommendations	73.4	76.8	75.0	
Saturated fatty acids (% of energy)				
Within the recommendations	3.1	0.0	1.7	0.182
Above the recommendations	96.9	100.0	98.3	
Polyunsaturated fatty acids (% of energy)				
Below the recommendations	93.8	96.4	95.0	
Within the recommendations	4.7	3.6	4.2	0.611
Above the recommendations	1.6	0.0	0.8	
Monounsaturated fatty acids (% of energy)				
Below the recommendations	7.8	5.4	6.7	0.591
Within the recommendations	92.2	94.6	93.3	
Cholesterol (mg)				
Within the recommendations	71.9	71.4	71.7	0.957
Above the recommendations	28.1	28.6	28.3	
Available carbohydrates (% of energy)				
Below the recommendations	60.9	69.6	65.0	0.319
Within the recommendations	39.1	30.4	35.0	
Dietary fibre (g)				
Below the recommendations	34.4	32.1	33.3	0.796
Within the recommendations	65.6	67.9	66.7	
Total water (g)				
Below the recommendations	70.3	69.6	70.0	0.936
Within the recommendations	29.7	30.4	30.0	

*P* – significance.

## Discussion

### Summary of the studies selected for comparison of dietary intake

As it was stated in the *Introduction*, no publications on dietary intakes of only 6-year-old children were found in the literature published after the year 2000. Therefore, to compare the results, studies which included 6-year-olds or children of approximate age were searched for. The bases which were searched included: EBSCOhost, PubMed, ScienceDirect. Also reports of national surveys published on the government websites of the United Kingdom [31] and the United States [32] were used. The summary of these studies is showed in Additional file 1: Table S1. This summary shows that the age groups which were the most similar to the age of the studied children were: the population of Spanish 6-7-year-olds [12,13], Cretan children aged 6.8 years [11] and 7-year-old English children [33]. In six out of twelve studies, the method of food record was applied: estimation using household measures was used in five studies [8-11,15,33] and weighed food record was used in one study [7]. The studies most frequently covered one day of intake (five out of twelve studies) [6,14-16,34]. All of the studies included intake of energy, however, in terms of nutrients which were analysed, the studies were diverse. Only in two studies [9,13], the percentages of children below, above or within the recommendations were presented.

### Energy intake

Energy intake was adequate in most of the studied 6-year-olds which was reflected in the highest percentage of children with healthy weight. Although the percentages of underweight and obese children were low, there was a substantial percentage of overweight children. It is highly unfavourable because in overweight children the risk of being overweight or becoming obese later in life is higher than in normal-weight children [35]. It is interesting that energy intake increased through all of the percentile categories, except for obesity. The relatively low energy intake observed in obese children is most probably due to underreporting of food intake by their parents. Although underreporting is little explored in children aged 6 years or less, it is well known that the rate of underreporting is higher in overweight subjects, compared to non-overweight, and the highest in the obese [36-39]. In the current study, the probability of underreporting by the preschool staff, who recorded children’s food intake during the stay in the preschool, is very low because of the high motivation and involvement of the staff along with the supervision of the author of the article.

The observed higher energy intake in boys is consistent with the results of the previous studies which reported significantly higher energy intake in boys compared to girls of various age and from various countries: in 4–5.6-year-olds from Belgium [9], in 7-year-olds from England [33], in 5-11-year-olds from France [15], in 4-5-year-olds from Greece [40] and in 7-9-year-olds from Portugal [34] and in 4-5-year-olds from Vietnam [41].

### Macronutrient intake

#### Protein

In comparison to the previously studied children, intake of energy from protein in the studied 6-year-olds was lower than in Belgian 4.5-6-year-olds [9], French 5-11-year-olds [15] and Greek children [11,40], and much lower than in Spanish 6-7-year-olds [12,13], Spanish 6-9-year-olds [14], Portuguese 7-9-year-olds [34] and Vietnamese 4-5-year-olds [41]. However, it was higher than in British [7] and Polish [6] 4-6-year-olds, and similar to energy from protein in the diets of American children aged 6–11 years and less than 6 years [16]. Intake of protein per kg of body weight in the studied 6-year-olds was lower than in Belgian 4.5-6-year-olds [10] and protein density was lower than in Cretan children [11].

Total protein intake in the studied 6-year-olds poses little risk of deficiency. However, there were substantial percentages of girls and boys whose intake of energy from total protein was above the recommended. Nevertheless, these percentages were much lower than in Belgian 4.5-6-year-olds (50.0% of girls and 56.5% of boys) [9].

Intake of animal protein (% of total protein) in the studied 6-year-olds was similar to that observed in the previously studied Polish 4-6-year-olds [6], Belgian 4–6.5-year-olds [10] and Vietnamese 4-5-year-olds [41]. It is recommended to reduce the intake of animal protein since its high intake is related to an increased diabetes risk [42], as well as to earlier pubertal onset which may contribute to a higher risk of breast cancer [43]. Moreover, it causes reduced intake of plant protein which is inversely related to the risk of ischaemic heart disease [44]. It is surprising that although the studied children attended preschools promoting health, their intake of total, animal and plant protein did not differ much from the intakes observed in other children.

#### Fat

Total fat intake in the studied 6-year-olds exceeded the recommendations and should be lowered. Former concerns that lowering energy from fat in children’s diets may cause decreased intakes and deficiencies of essential nutrients, and thus poor growth [45], have been dispelled by the results of many intervention and longitudinal studies on children of various age [46-49]. On the contrary, it is emphasised that energy from fat in children’s diets should not exceed 30% because of the benefits to lipid profile [47,50] as well as reduced risk of cardiovascular diseases and cancer, not only in the short term but also in adulthood [51].

Although the studied 6-year-olds exceeded the recommendations on fat intake, energy from fat was the lowest compared to children from other European countries: France [15], Great Britain [7,8,33], Greece [11,40], Portugal [34] and Spain [12-14]. It is important to note that intake of energy from fat was the highest in Greek children [40] and Cretan children [11], as well as Spanish 6-7-year-olds [12,13], reaching 40% and more energy from this macronutrient. Only in Belgian 4–6.5-year-olds [9] and Polish 4-6-year-olds [6], intake of energy from fat was lower than in the studied 6-year-olds, but only Belgian children met the recommendations [9]. In comparison to children from outside Europe, intake of energy from fat in the studied children was similar to the intake observed in American children aged 6–11 years and less than 6 years [16], but it was much lower than in Vietnamese 4-5-year-olds [41].

The structure of fatty acid intake was also unfavourable. It was characterised by excessive intake of saturated fatty acids, higher even than monounsaturated fatty acid intake, along with inadequate intake of polyunsaturated fatty acids. It is especially disconcerting in case of boys, since it is well recognised that males are at higher risk for atherosclerosis than females and the studied boys’ intakes of both total fat and saturated fatty acids (g, % of energy, g/1000 kcal) were significantly higher in comparison to girls. Moreover, inadequate intake of polyunsaturated fatty acids observed in almost all of the studied 6-year-olds may have adverse effect on their neurodevelopment [52].

Intakes of fatty acids in children of various age from other countries were usually similar to those observed in the current study. Excessive intake of energy from saturated fatty acids was observed also in the diets of children in Belgium [9], France [15], Great Britain [7,8,33], Greece [11,40], Portugal [34], Spain [12,14] and the United States [16], as well as in the previously studied Polish children [6]. Intake of energy from polyunsaturated fatty acids was lower than the recommended also in the diets of Belgian 4–6.5-year-olds [9], British children [8,33], Cretan children [11], Polish 4-6-year-olds [6], Portuguese 7-9-year-olds [34] and Spanish 6-9-year-olds [14], and adequate only in the diets of Spanish 6-7-year-olds [12]. Intake of energy from monounsaturated fatty acids in the studied 6-year-olds was much lower than in Cretan children [11], Spanish 6-7-year-olds [12] and Spanish 6-9-year-olds [14], lower than in Polish 4-6-year-olds [6] and Portuguese 7-9-year-olds [34], similar to British children [8,33], but higher than in Belgian 4–6.5-year-olds [9]. Nutrient densities of fatty acids in the studied children’s diets were lower than in Cretan children [11], especially for saturated and monounsaturated fatty acids.

Although cholesterol intake in the studied 6-year-olds was in accordance with the recommendations, it was higher than in American children aged 6-11-years and less than 6 years [16] and in Polish 4-6-year-olds [6], and much higher than in Belgian 4–6.5-year-olds [9]. However, it was lower than in Spanish 6-7-year-olds [12] and Spanish 6-9-year-olds [14] who exceeded the recommendations. Cholesterol density was higher than in Cretan children [11], but lower than in Spanish 6-7-year-olds [12].

Despite not exceeding the recommendations on cholesterol intake by the studied children, density of this nutrient, along with the adverse structure of fatty acid intake, need urgent intervention. Otherwise, unfavourable structure of fatty acid intake will soon be accompanied by excessive cholesterol intake due to the inevitable increase of energy intake as children grow. It is surprising that although the children attended preschools aimed at promoting health, their intakes of energy from fat and fatty acids were so unfavourable. These findings confirm unfavourable food habits observed in the previous studies on Polish preschoolers [53,54]. Moreover, these findings are similar to those obtained in other populations of children in Europe and show the need to work out a common educational programme to improve nutrition in young European children taking into account food habits which are specific to the tradition or food supply of each country.

#### Carbohydrates

Intake of available carbohydrates in the studied 6-year-olds was below the recommended. However, it is worth noting that carbohydrate content of foods in Polish food composition tables [20] was calculated by difference, that is by subtracting the content of moisture, protein, fat, ash and alcohol from the total weight of the food [21]. Intake of carbohydrates was reported to be 14% higher when measured by difference compared to carbohydrates measured directly (direct analysis of carbohydrate components and summation to obtain a total carbohydrate value) [21]. Therefore, it is probable that intake of carbohydrates in the studied 6-year-olds was in fact even more below the recommendations than it can be observed from the obtained results.

Due to the differences in methodology, it is difficult to compare the results with the results of other studies. For sure, intake of energy from carbohydrates in the studied 6-year-olds was lower than in Polish 4-6-year-old children studied by Szponar et al. [6] who used the same method of carbohydrate determination. In comparison to British 4-6-year-olds [7], British 4-10-year-olds [8], British 7-year-olds [33] and French 5-11-year-olds [15], intake of energy from carbohydrate in the studied 6-year-olds was higher. However, the aforementioned studies on British and French children used McCance and Widdowson’s ‘The Composition of Foods’ in which carbohydrate content was obtained by direct analysis [21]. Therefore, if in case of French 5-11-year-olds [15] the difference in energy from carbohydrates is 11.3% compared to the studied 6-year-olds, the observed difference is mainly due to different methodology and so the intake is similar. And if in case of British children this difference is from 2.3% [8] to 4.0% [33], it is probable that the intake of energy from carbohydrates by the studied 6-year-olds was in fact lower than in British children. Surely, intake of energy from carbohydrates in the studied 6-year-olds was lower than in Belgian 4–6.5-year-olds [9] whose intake was 54.87% in girls and 54.19% in boys and was determined using McCance and Widdowson’s The Composition of Foods. The lowest intake of energy from carbohydrates was reported in Spanish 6-7-year-olds [12,13], only 38.3%, however, the method of carbohydrate determination in Spanish food composition tables was not available to the author.

Intake of sucrose in the studied 6-year-olds seems to be high. It is recommended to reduce intake of all monosaccharides and disaccharides, that is also sucrose, added to foods by the manufacturer, cook or consumer, as well as sugars naturally present in honey, syrups and fruit juices to less than 10% of energy [25]. In the studied 6-year-olds the intake of energy only from sucrose was almost twice higher than the WHO recommendations for all added monosaccharides and disaccharides. These findings reflect the adverse habit of adding a lot of sugar to tea and other beverages, which is very popular in the studied region, as well as the adverse habit of snacking on sweets between the main meals. A study on Polish 5-6-year-olds showed that more than 50% of the children snacked on sweets twice or more times a day [53]. Besides, it is well recognised that preferences for sweet taste are innate and typical of infants and young children irrespective of gender [55,56] and probably this was also an important factor of high intake of sucrose in the studied 6-year-olds.

High intake of sucrose in the studied 6-year-olds is very unfavourable. The studies showed that an increase in sucrose intake increases triacylglycerol concentration [57] which is the risk factor for atherosclerosis. Moreover, high intake of added sugars in children is associated with lower intakes of micronutrients [58-61] and with lower intakes of important food groups such as grains, vegetables, fruits, and dairy [58,60,61]. Most of the staff who worked in the studied preschools and most of the studied children’s parents knew that high sucrose intake increases dental caries [62,63] and that sugar intake should be limited because it does not provide any additional nutrients in children’s diets [64,65]. Therefore, it is surprising to find high sucrose intake in children who attended preschools aimed at promoting health. Most probably food habits were stronger than knowledge.

It is interesting that intake of sucrose (% of energy, g/1000 kcal) in the studied girls was significantly higher than in boys. This seems to reflect female higher preferences for sweet taste which are typical in teenagers and adult women [66-68].

In other studies, intake of sucrose was not analysed. In British 4-10-year-old children [8] and British 7-year-olds [33], intake of non-milk extrinsic sugars was reported to exceed the recommendations. In the study on Belgian 4–6.5-year-olds [9], Spanish 6-7-year-olds [12,13] and Portuguese 7-9-year-olds [34], intake of energy from simple carbohydrates was reported to be high, however, only Moreira et al. [34] defined the term ‘simple carbohydrates’ as all monosaccharides and disaccharides added to foods by the manufacturer, cook or consumer, as well as sugars naturally present in honey, syrups and fruit juices.

No recommendations on starch intake are available. Intake of energy from this macronutrient in the studied 6-year-olds was similar to that reported in British 7-year-olds [33] and slightly lower than in British 4-10-year-olds [8]. In other studies, intake of energy from complex carbohydrates was reported. In French 5-11-year-olds [15], it was similar to intake of energy from starch in the studied 6-year-olds, whereas it was slightly lower in Belgian 4–6.5-year-olds [9]. Very low intake of energy from complex carbohydrates was found in Spanish 6-7-year-olds, only 17.8% [13].

Dietary fibre intake in most of the studied 6-year-olds was in accordance with the recommendations and was similar to the intake reported in Polish 4-6-year-olds [6], in Belgian 4–6.5-year-olds [9] and in Spanish 6-9-year-olds [14] but it was lower than in Spanish 6-7-year-olds [13] and Portuguese 7-9-year-olds [34]. Dietary fibre density in the studied children’s diets was higher than in Cretan children [11] but similar to dietary fibre density in the diets of Belgian 4–6.5-year-olds [9]. In British 4-10-year-olds [8] and British 7-year-olds [33], intake of non-starch polysaccharides was analysed so the comparison is not possible.

#### Water

There is a concern that total water intake in the studied 6-year-olds may be inadequate. Low intake of water is unfavourable since water is not only essential for day-to-day health [69] but may also play a role in the prevention of chronic diseases [70]. It is probable that parents underestimated the role of this macronutrient because they often hear in the Polish mass media about the importance of protein, vitamins or minerals to the health of their children, but rarely attention is drawn to the importance of water. However, it cannot be excluded that some parents forgot about reporting their children’s water intake despite having been asked by the author to do so. It is also possible that preschool staff failed to fully control the children who had access to water during preschool hours. Despite the fact that the author and the preschool staff asked the children to inform the teacher each time they would like to drink water, it is possible that not all children remembered about it.

Water intake was analysed only in one study on Belgian 4–6.5-year-olds [9] and was reported to be low. Also in the studies on older children and adolescents, information on water intake was provided by only a few studies [71]. This issue needs further studies, since providing information on water intake in children is of great importance to public health and to working out nutritional education programmes for the societies.

## Conclusions

In conclusion, many adverse characteristics of the children’s diets were observed, mainly the excessive intake of total fat, saturated fatty acids and sucrose, along with inadequate intake of polyunsaturated fatty acids, available carbohydrates, and starch. These tendencies are common to the diets of children of similar age in other European countries and show the need to work out a common educational programme to improve nutrition in young European children taking into account food habits which are specific to the tradition or food supply of each country. From the public health point of view, it is important to provide the lacking information about the intake of animal and plant protein, as well as about water intake in young children in Europe.

## Competing interests

The author declares that she has no competing interests. Financial support was received from the Polish Ministry of Science and Higher Education.

## Pre-publication history

The pre-publication history for this paper can be accessed here:

http://www.biomedcentral.com/1471-2431/14/197/prepub

## Supplementary Material

Additional file 1: Table S1 The summary of the studies on dietary intake, which included 6-year-olds or children of approximate age, used in the section *Discussion* (studies showed in alphabetical order of the country). Click here for file
