# Supplementary material for: Dietary intake in 6-year-old children from southern Poland: part 1 - energy and macronutrient intakes
Source: BMC Pediatr. 2014 Aug 3;14:197. doi: 10.1186/1471-2431-14-197 (PMC4126390; doi:10.1186/1471-2431-14-197)
Supplement: Additional file 1: Table S1 — The summary of the studies on dietary intake, which included 6-year-olds or children of approximate age, used in the section Discussion (studies showed in alphabetical order of the country). [file 1471-2431-14-197-S1.doc]

| **Additional file 1: Table S1: The summary of the studies on dietary intake, which included 6-year-olds or children of approximate age, used in the section *Discussion* (studies showed in alphabetical order of the country)** | | | | | |
| --- | --- | --- | --- | --- | --- |
| **Reference** | **Country/**  **region** | **The studied population; the name of the study (if provided)** | **Subgroup/**  **subgroups selected for comparison** | **The method of assessing dietary intake** | **Energy and macronutrients analysed** |
| Huybrechts & De Henauw, 2007 [9];  Lin et al., 2011 [10] | Belgium, Flanders | a representative sample of 2.5-6.5-year-old children | 4-6.5-year-olds | a 3-day estimated food record | energy (kJ, kcal); macronutrients expressed in g and as % of energy: total protein, animal protein, plant protein, fat, carbohydrates; macronutrients expressed as % of energy: SFA, PUFA, MUFA, complex carbohydrates, simple carbohydrates; protein (g/kg); cholesterol (mg); fibre (g, g/1000 kcal); water (ml, ml/kg); the percentages of the children with usual intakes below or above dietary reference intakes |
| Glynn et al., 2005 [33] | Southwest England, the county of Avon | 7-year-old children; the Avon Longitudinal Study of Parents and Children (ALSPAC) | – | a 3-day estimated food record | energy (MJ); macronutrients expressed in g and as % of energy: protein, fat, SFA, PUFA, MUFA, carbohydrate, starch, total sugar, non-milk extrinsic sugar; non-starch polysaccharides (g) |
| Maillard et al., 2000 [15] | France; two towns in northern France | 5-11-year-old children; the Fleurbaix Laventie Ville Santé Study | – | a 1-day estimated food record | energy (kcal); macronutrients expressed as % of energy: protein, fat, SFA, carbohydrates, complex carbohydrates |
| Great Britain Office for National Statistics Social Survey Division, 2000 [7] | Great Britain | 4-18-year-old children and adolescents; National Diet and Nutrition Survey: young people aged 4-18 years | 4-6-year-olds | a 7-day weighed food record | energy (kcal); macronutrients expressed in g and as % of energy: protein, fat, carbohydrates; SFA (% of energy); cholesterol (mg) |
| Manios, 2006 [40] | Greece | a representative sample of Greek toddlers and preschool children aged 1-5 years;  the Growth, Exercise and Nutrition Epidemiological Study in preSchoolers (GENESIS) | above 4- to 5-year-olds | 3 days: weighed food record (during nursery hours) and 24-hour recall or food diaries (outside nurseries) | energy (MJ, kcal); macronutrients expressed in g and as % of energy: protein, fat, SFA, carbohydrates |
| Smpokos et al., 2013 [11] | Greece, Crete | children aged 5.7-7.6 years from two representative samples: 1992/93 and 2006/07 | the sample studied in 2006/07 – mean age 6.8 years | a 3-day estimated food record | energy (kJ); macronutrients expressed as g/1000 kcal and % of energy: protein, fat, SFA, PUFA, MUFA, carbohydrates; cholesterol (mg/1000 kcal), dietary fibre (g/1000 kcal) |
| Szponar et al., 2003 [6] | Poland | a representative sample of people aged 1 year and older | 4-6-year-olds | a single 24-hour dietary recall | energy (kcal); macronutrients expressed in g and as % of energy: protein, fat, SFA, PUFA, MUFA, total carbohydrate; animal and plant protein (g, % of total protein); cholesterol (mg); dietary fibre (g) |
| Moreira et al., 2005 [34] | Portugal | 7-9-year-old children | – | a single 24-hour dietary recall | energy (kcal); macronutrients expressed as % of energy: protein, fat, SFA, PUFA, MUFA, total carbohydrates, sugars; protein (g/kg); fibre (g) |
| Rodríguez-Artalejo et al., 2002 [12];  Royo-Bordonada et al., 2003 [13] | Spain, four cities: Cadiz, Murcia, Madrid, Orense | 6-7-year-old children | – | food frequency questionnaire | energy (kcal); macronutrients expressed in g and as % of energy: protein, fat, SFA, PUFA, MUFA, total carbohydrates, complex carbohydrates, simple carbohydrates; cholesterol (mg, mg/1000 kcal); fibre (g); the percentages of children complying with nutritional goals set for the Spanish population |
| Serra-Majem et al., 2006 [14] | Spain | people aged 2-24 years; the enKid Study | 2-5-year-olds;  6-9-year-olds | a single 24-hour dietary recall and a food frequency questionnaire | energy (kJ, kcal); macronutrients expressed in g and as % of energy: protein, fat, SFA, PUFA, MUFA, carbohydrate; cholesterol (mg, mg/1000 kJ); fibre (g, g/1000 kJ) |
| Bates et al., 2012 [8] | all four  countries of the United Kingdom | a representative sample of people aged 1.5 years and older; the National Diet and Nutrition Survey: the Rolling Programme (2008/2009 – 2010/2011) | 4-10-year-olds | a 4-day estimated food record | energy (MJ, kcal); macronutrients expressed in g and as % of energy: protein, fat, SFA, PUFA, MUFA, total carbohydrate, starch, total sugars, intrinsic and milk sugars, non-milk extrinsic sugars; non-starch polysaccharide (g) |
| Wright et al., 2003 [16] | USA | people of all ages; the National Health and Nutrition  Examination Survey 1999-2000 | less than 6-year-olds;  6-11-year-olds | a single 24-hour dietary recall | energy (kcal); macronutrients expressed as % of energy: protein, fat, SFA, carbohydrate; cholesterol (mg) |
| Huynh et al., 2008 [41] | Vietnam | 4-5-year-old children | – | food frequency questionnaire | energy (kcal); macronutrients expressed in g and as % of energy: total protein, animal protein, plant protein, fat, animal fat, vegetable fat, carbohydrate, free sugar |
| SFA – saturated fatty acids; PUFA – polyunsaturated fatty acids; MUFA – monounsaturated fatty acids. | | | | | |
